# Water Orientation at the Calcite-Water Interface

**DOI:** 10.1021/acs.jpclett.1c01729

**Published:** 2021-08-05

**Authors:** Hagen Söngen, Simon J. Schlegel, Ygor Morais Jaques, John Tracey, Saman Hosseinpour, Doyk Hwang, Ralf Bechstein, Mischa Bonn, Adam S. Foster, Angelika Kühnle, Ellen H.G. Backus

**Affiliations:** † Physical Chemistry I, Faculty of Chemistry, Bielefeld University, Universitätsstrasse 25, 33615 Bielefeld, Germany; ‡ Max Planck Institute for Polymer Research, Ackermannweg 10, 55128 Mainz, Germany; § Department of Physical Chemistry, University of Vienna, Währinger Strasse 42, 1090 Vienna Austria; ∥ Department of Applied Physics, Aalto University, Helsinki, FI-00076, Finland; ⊥ Nano Life Science Institute (WPI-NanoLSI), Kanazawa University, Kanazawa 920-1192, Japan

## Abstract

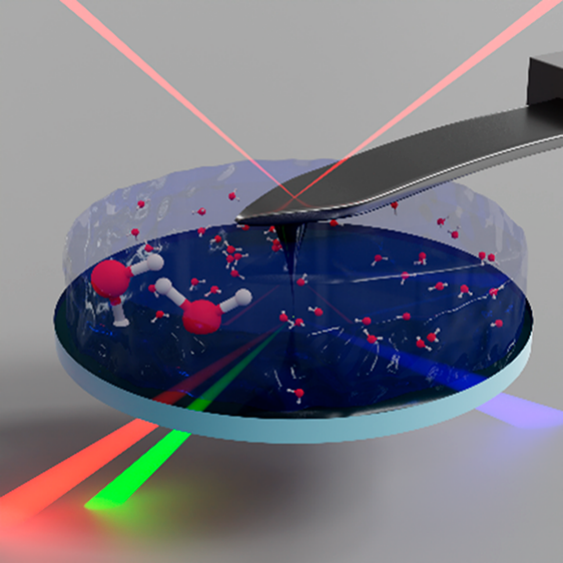

Mineral-water interfaces play an important role in many natural as well as technological fields. Fundamental properties of these interfaces are governed by the presence of the interfacial water and its specific structure at the surface. Calcite is particularly interesting as a dominant rock-forming mineral in the earth’s crust. Here, we combine atomic force microscopy, sum-frequency generation spectroscopy, and molecular dynamics simulations to determine the position and orientation of the water molecules in the hydration layers of the calcite surface with high resolution. While atomic force microscopy provides detailed information about the position of the water molecules at the interface, sum-frequency generation spectroscopy can deduce the orientation of the water molecules. Comparison of the calcite-water interface to the interfaces of magnesite-water, magnesite-ethanol, and calcite-ethanol reveals a comprehensive picture with opposite water orientations in the first and second layer of the interface, which is corroborated by the molecular dynamics simulations.

Mineral-water interfaces^1^ are ubiquitous in nature and play an important role in a wide variety of fields, including, e.g., biochemistry, geochemistry,^2^ and catalysis. An understanding of interfacial processes in these fields thus requires elucidating the spatial arrangement and orientation of the water molecules at the mineral interface. In this context, calcite, the most stable form of calcium carbonate (CaCO_3_), is of great importance as it represents the most abundant carbonate in the earth’s crust. Consequently, the arrangement of water at the most stable calcite cleavage plane, the calcite (10.4) surface, has been studied intensively in the past, both experimentally^3−9^ and theoretically:^3−5,10−15^ Using three-dimensional atomic force microscopy (3D AFM),^16^ the hydration structure at the interface can be directly mapped.^6^ These AFM data have revealed a so-called “checkerboard pattern”^6^ of the water molecules at the calcite-water interface, meaning that areas of bright contrast in AFM frequency shift alternate with areas of dark contrast, creating a pattern that is similar to a checkerboard. Comparing the experimental AFM data from frequency modulation AFM with data from molecular dynamics simulations (MD) has revealed a close relationship between the frequency shift data and the calculated water density.^3,17,18^ Therefore, 3D AFM has developed into a powerful tool to study not only 2D spatial arrangement of the hydration structure but also the 3D arrangement at the interface. For instance, 3D AFM is nowadays used to study dissolution at calcite step edges^19^ as well as point defects in the crystal surface.^4^ Complementary information is obtained from X-ray reflectivity (XRR) data,^5,7−9^ which provides insight into the averaged spatial position of the interfacial atoms with utmost accuracy and precision.

On the basis of these complementary studies, a detailed picture can be drawn, representing our current understanding of the ordered hydration structure of water at the calcite (10.4) surface. Vertical cuts through 3D AFM data (i.e., *xz*-slices with the surface at the bottom) show the characteristic checkerboard pattern of dark and bright features with the bright features associated with high water density. A typical *xz*-slice taken at the calcite-water interface is given in Figure 1a. The checkerboard arrangement has been confirmed by MD simulations,^3,12^ and corresponding data for the calcite-water interface are shown in Figure 2a. This figure shows updated simulations (see SI for details), providing the possibility for a detailed analysis of the angular distributions of water molecules at the hydration layer and the hydrogen bonding distance (see below). The AFM (Figure 1a) and MD data (Figure 2a) show that in the first layer, the water is situated above the surface calcium atom (bright features close to the surface), while the second layer consists of water molecules above the surface carbonate groups. The AFM data reveal the average density of the water molecules with high spatial resolution, but do not provide the orientation of water. The same is true for XRR data, which are insensitive against hydrogen. Thus, the orientation of the water molecules has so far been deduced from calculations only.^15^ MD and density-functional theory^11,12,15^ simulations suggest that the water molecules in the first layer have their oxygen atoms pointing toward the surface to allow for electrostatic binding to the surface calcium cation, while the water molecules in the second layer form a hydrogen bond to the protruding oxygen atom of the surface carbonate group.

**Figure 1:**
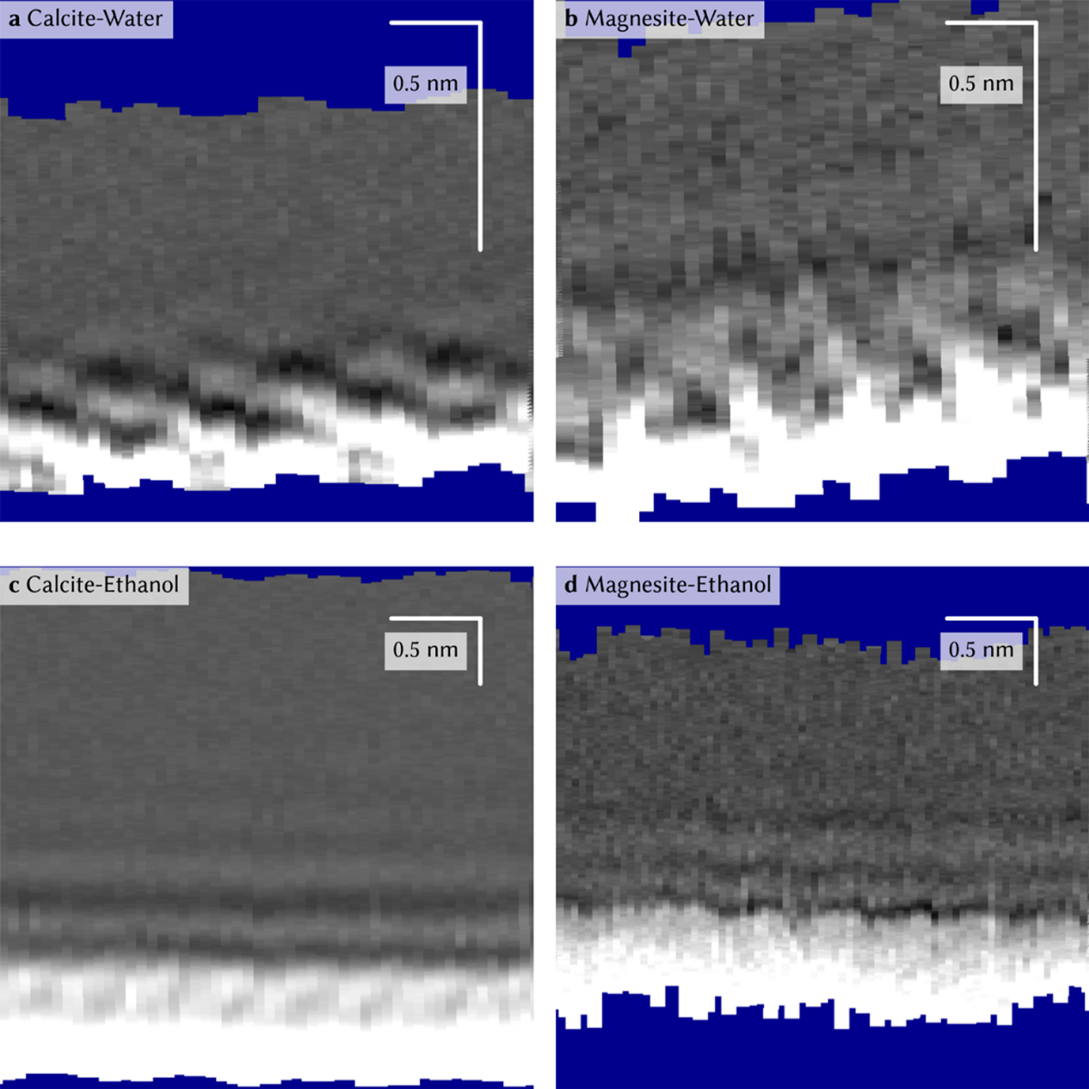
Vertical (*xz*) slices extracted from 3D AFM data. Details of data acquisition and analysis can be found in a previous publication.^12^ (a) The checkerboard hydration structure of water above the calcite (10.4) surface (at the bottom of the plot). Similar data have been published before.^3,6,20^ On the basis of the solvent-tip approximation,^17,18^ the bright features are associated with high water density. (b) The hydration structure at the water-magnesite (10.4) interface for comparison, revealing a qualitatively similar checkerboard picture. Solvation structure of ethanol at the (c) ethanol-calcite (10.4) and (d) ethanol-magnesite (10.4) interface. The ethanol data have been previously published in the literature.^21^

**Figure 2:**
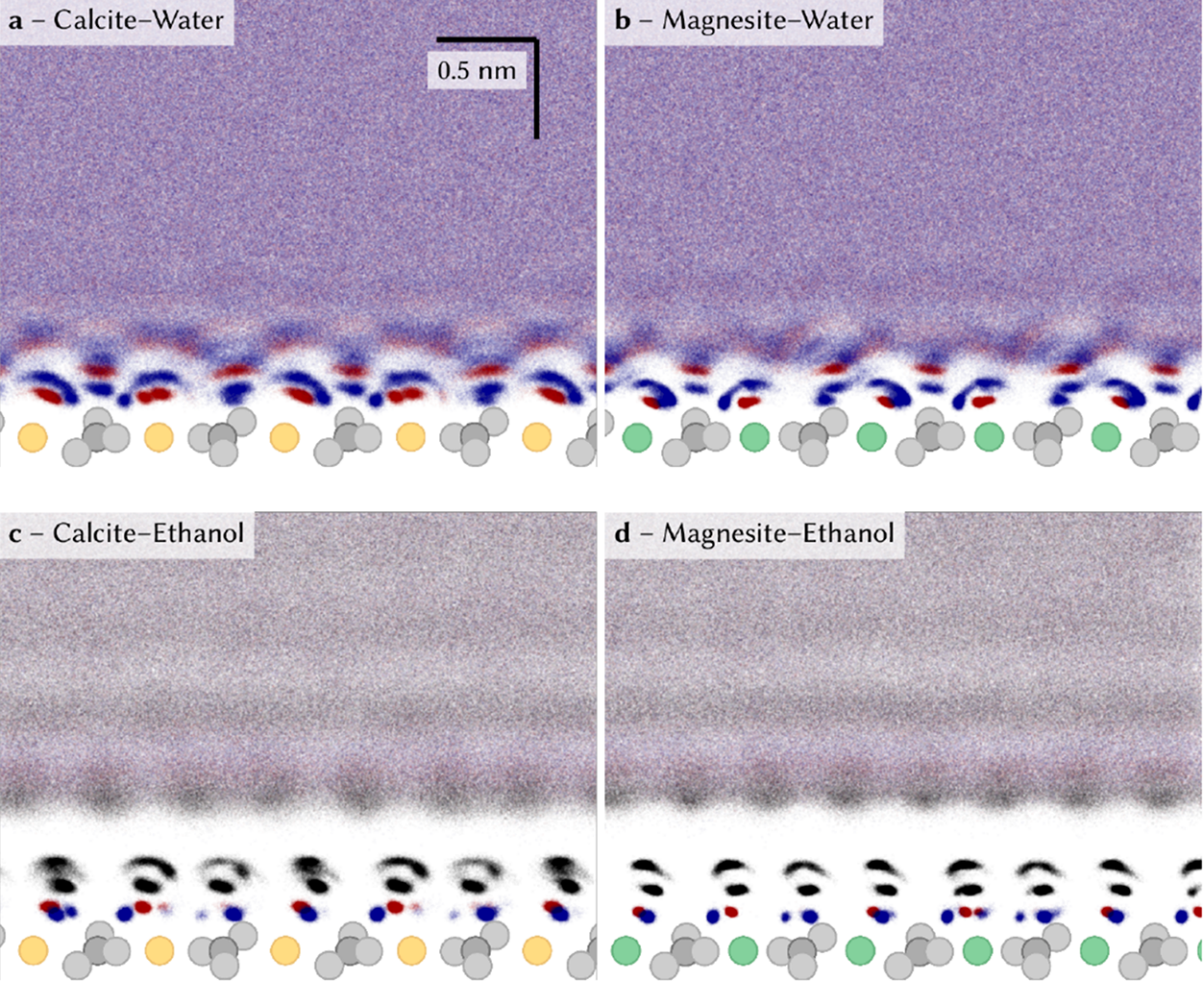
Molecular dynamics simulations for pristine surfaces showing the atomic positions of the respective atoms along with a density plot. Calcium and magnesium ions are represented in yellow and green, respectively. The oxygen and hydrogen atoms of the water/ethanol molecules are marked in red and blue, respectively. The CH groups of the ethanol molecules are represented in black. The data show (a) the calcite-water and (b) magnesite-water interfaces. For comparison, the same data are reproduced for (d) the calcite-ethanol and (d) magnesite-ethanol interfaces from the literature.^21^ The double red density regions for the water oxygen atoms in the first hydration layer are caused by two oxygen atoms (each from two adjacent carbonate groups) being within hydrogen bond distance. The carbonate oxygen with which the water molecule interacts is prone to vary throughout the simulations.

While the theoretical picture is very convincing, experimental verification of the water orientation is at present still lacking. Inherently surface-sensitive sum-frequency generation (SFG) spectroscopy is the method that can provide specifically this information.^22,23^ In this method, an infrared laser beam in resonance with a molecular vibration, in our case the O–H stretch vibration, is overlapped in space and time with a visible laser beam. At the interface, the sum-frequency light of the two incoming beams is generated. In the electric dipole approximation, the generation of sum-frequency signals is forbidden in centrosymmetric media. The appearance of an SFG signal thus means that the molecules are preferentially aligned, breaking centrosymmetry. The SFG signal is enhanced at resonance with the molecular vibration.

Here, we combine the complementary information from SFG spectroscopy and AFM to provide a comprehensive picture of the hydration structure at the buried mineral-water interface. From this interplay, we obtain experimental evidence for an opposite orientation of the water molecules in the first and second layer, which is perfectly corroborated by the MD results.

The experimental data are obtained by bringing the minerals (calcite and magnesite) in contact with liquid water (see Figure S1). Experimental details can be found in the Supporting Information.

The SFG spectrum of the calcite-water interface (Figure 3a) in the O–H stretch region shows, despite a very long acquisition time, only a very small signal at 3400 cm^–1^, barely exceeding the noise level for both pure water and water with 1 mM NaCl (see SI). The very weak signal could originate from either the calcite substrate or from water. Control experiments, with D_2_O in contact with the mineral (gray curve in Figure 2a), show that the small signal at 3400 cm^–1^ is coming from water and not from the substrate, as the signal disappears upon adding D_2_O. The SFG signal intensity scales with the number of ordered molecules at the interface and is proportional to their degree of ordering. Given the high order of water molecules detected in the AFM picture (see above), the very weak SFG signal is surprising.

**Figure 3:**
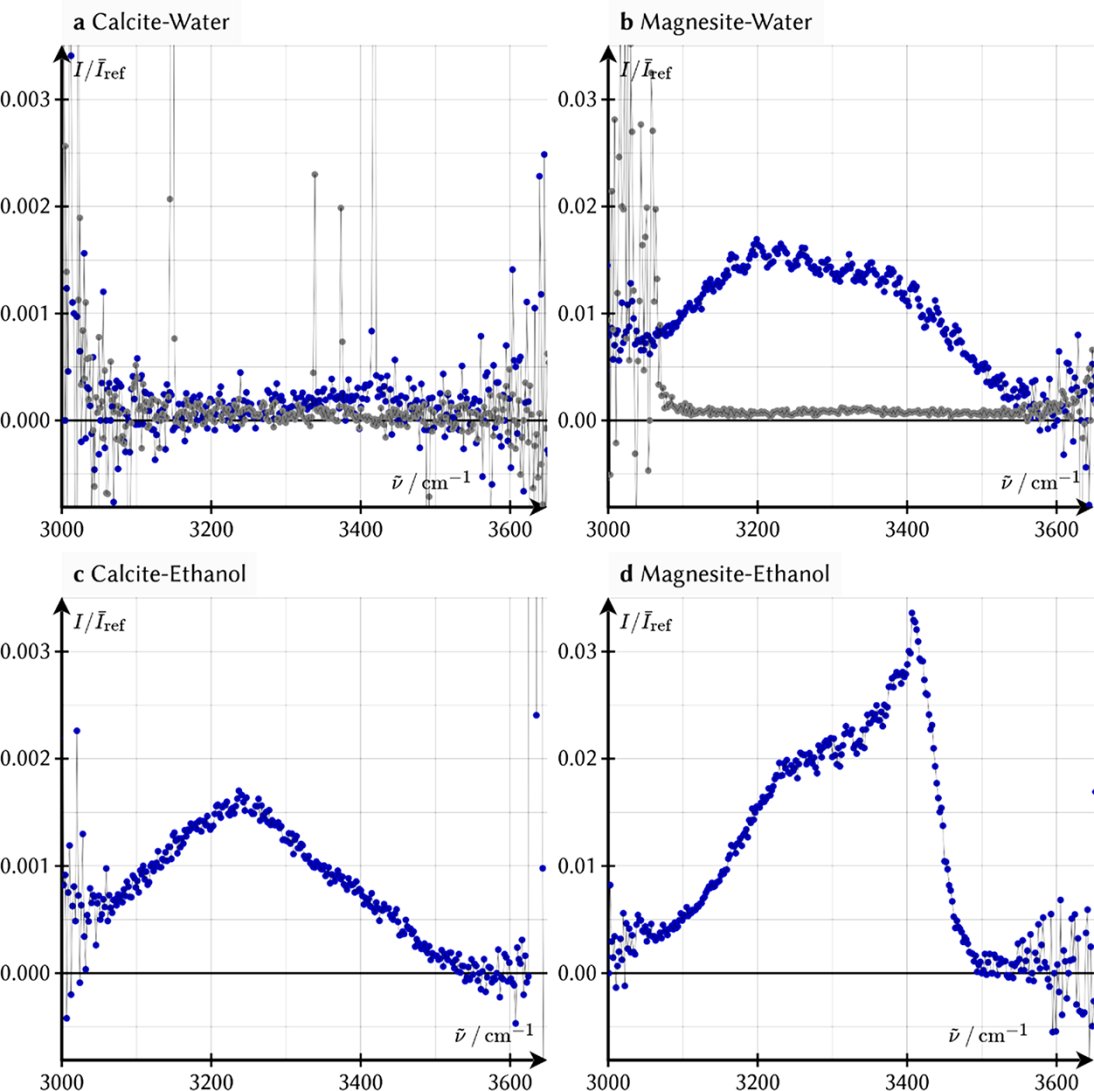
SFG intensity in ssp polarization (SFG and visible s-polarized; IR p-polarized) as a function of the IR frequency for the calcite-water (a) (H_2_O: blue data; D_2_O: gray data), (b) the magnesite-water (H_2_O: blue data; D_2_O: gray data), (c) the calcite-ethanol, (d) and the magnesite-ethanol interface. The SFG spectra are normalized by the SFG signal from a calcite-gold and magnesite-gold interface, respectively. The etaloning on the SFG spectrum in panels b and d originates from defects in the magnesite crystal. Please note that the 10-fold difference in the *y*-axis between calcite and magnesite.

A potential explanation for the absence of a clear SFG signal, despite the observed high lateral order, could be the unique arrangement of the water molecules with opposite orientations in the adjacent layers resulting in zero sum-frequency signal. Such an arrangement is indeed in line with the results from our MD simulations (see SI for simulation details), as apparent from Figure 2a. MD simulation results for vertical slices of the calcite water interface show that the water molecules in the first layer have their oxygen atom above the surface calcium cation, while the water molecules in the second layer form a hydrogen bond with the protruding oxygen atom of the surface carbonate group. As detailed in Figure 4a, the water dipoles in the first and second layer have opposite orientation as the angular distribution with respect to the surface normal for these two layers is more or less symmetric around 90 deg. In such a geometry, the water SFG signal from the two layers could indeed cancel each other out. The cancellation also indicates, that apparently, the vibrational frequency for water in the first and second hydration layer is similar, suggesting that they have similar hydrogen bond strength.

**Figure 4:**
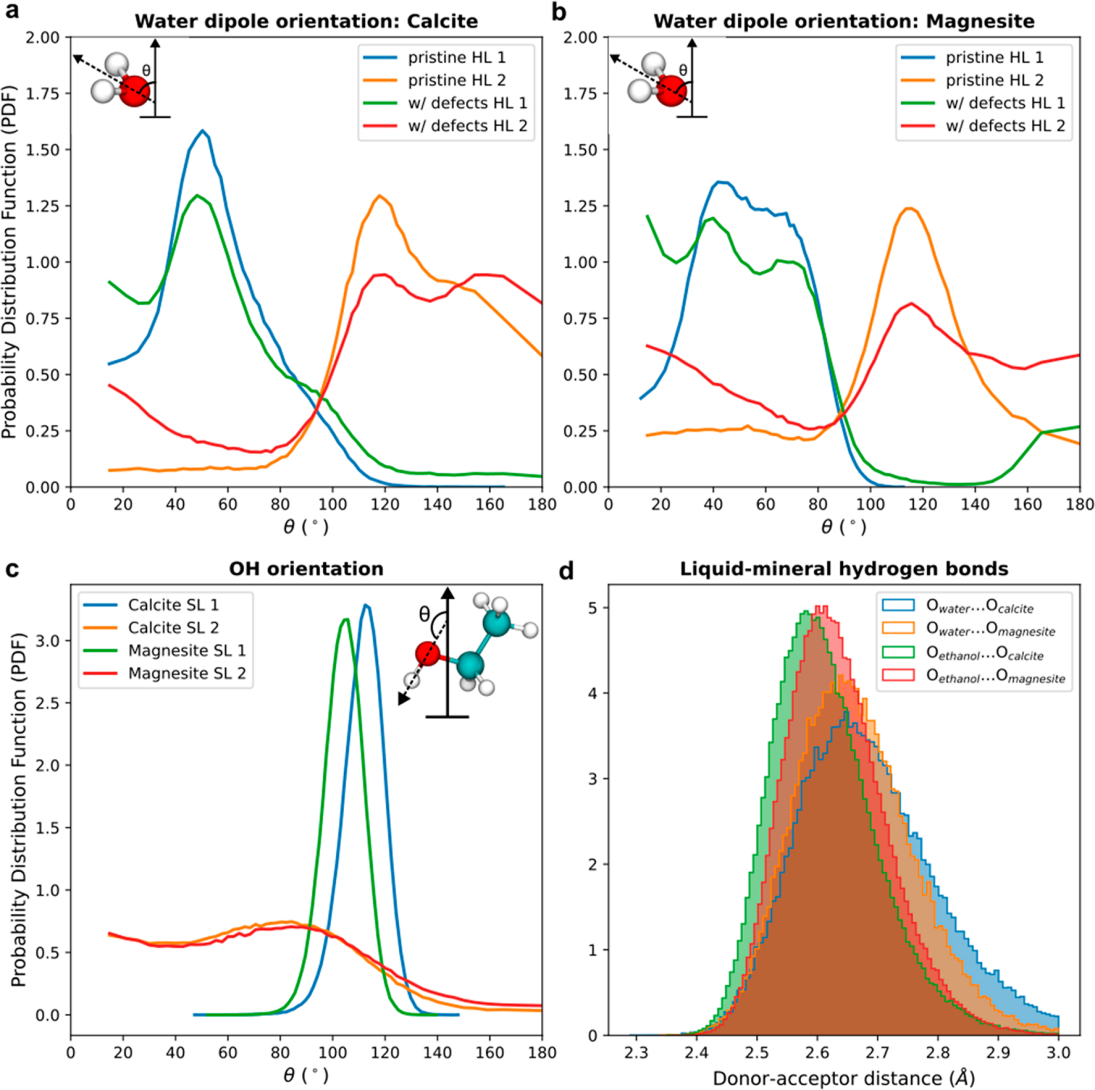
Water dipole orientation for (a) calcite/water and (b) magnesite/water in the first and second hydration layer (HL) for pristine surfaces as in Figure 2, and surfaces with defects. The dipole orientation angle in relation to the surface normal is defined as depicted in the insets. (c) Orientation of O–H bonds from the ethanol molecules for both calcite/ethanol and magnesite/ethanol interface in the first two solvation layers (SL). The inset shows the orientation angle in relation to the surface normal. (d) Histogram of the hydrogen bond distance between oxygen from water/ethanol to the oxygen from calcite/magnesite on the first HL and SL.

To test this hypothesis, we conducted SFG experiments on the structurally very similar mineral substrate magnesite. On the basis of the observed checkerboard structure with AFM (Figure 1b), the MD vertical slice (Figure 2b), and the opposite water orientation found in MD for layer one and two (Figure 4b), we would expect a similarly weak SFG signal. Surprisingly, as evident from Figure 3b, approximately 2 orders of magnitude larger SFG signal is observed at the magnesite-water interface, extending from ∼3100 to ∼3500 cm^–1^. As detailed in the SI, this difference in intensity does not originate from a difference in the Fresnel factors, i.e., the local field factors at the interface. However, as is clear from Figure S3, depicting the SFG signal for magnesite in contact with aqueous solutions of various NaCl concentrations, the intensity of the SFG signal depends strongly on the ion concentration. For higher ion concentration the SFG signal diminishes significantly. This observation is in line with refs.^24−27^ and can be explained by screening of interfacial charges upon adding salt to the aqueous solution. Apparently, an electric field builds up at the magnesite interface upon being in contact with water, either due to the presence of defects in the crystal or due to the dissolution of the mineral.^28^ X-ray photoelectron spectroscopy (XPS) results (see SI) indicate a less pure sample, i.e., potentially more defects, for the magnesite sample compared to the calcite sample. These defects could be responsible for the surface charge on magnesite. As a result, we conclude that the strong SFG signal arises mainly from the interfacial charges breaking the centrosymmetry in the bulk either by orienting the water molecules and/or polarizing them.^29^ In that case, the main part of the signal comes from the bulk of water.^24^ Of course, we cannot exclude a small contribution from the first and second layer to the signal, especially because the MD simulations shown in Figure 4b on magnesite with defects (cation and a carbonate group removed) showed that the distribution of the dipole orientation changes noticeably, resulting in a less pronounced orientation distribution.

Another way to test the hypothesis that the small SFG signal for calcite originates from the oppositely oriented water molecules within the first and second layer is to break this specific molecular alignment by exchanging water with ethanol. The interaction of ethanol with magnesite and calcite has been studied in ref (21) using both AFM (Figure 1c,d) and MD (Figure 2c,d). We know from these MD results that ethanol on both magnesite and calcite in the first layer is oriented with the OH group pointing to the surface. The second layer points with the hydrophobic side to the surface, while the third layer again has the OH group toward the interface. As the first layer is significantly more ordered than the other layers, a large SFG signal is expected. Accordingly, the dipole orientation of the OH groups in the first and second layers cannot cancel each other out in this case, as shown in the angular distribution for these two layers depicted in Figure 4c. The angular distribution of the first solvation layer is well-defined between 100 and 120 degrees, while the second solvation layer has a much broader distribution.

Indeed, Figure 3c,d depicts a clear SFG signal for the calcite-ethanol and magnesite-ethanol interfaces, the latter being an order of magnitude more intense than the former. This clear presence of the signal for the ethanol case, as opposed to the water case for calcite, demonstrates that breaking the symmetry results in an SFG signal. We thus conclude that for the calcite-water interface, the water molecules in the first and second layers are oppositely oriented and have the same vibrational frequency. Moreover, the spectrum for the magnesite-ethanol interface shows a double peak with signals around 3200 and 3400 cm^–1^. To obtain information about the relative orientation of the two O–H ensembles resembled by these signals, we describe the SFG data with the Lorentzian line shape model (see Methods Section in the Supporting Information), including two peaks and a small nonresonant contribution. The best description is obtained assuming two oppositely oriented Lorentzian peaks (see Supporting Information). These two peaks thus represent O–H groups of ethanol pointing with the dipoles in opposite directions.

To assign the two peaks, we compare the SFG spectrum to the spectrum of the ethanol-air interface; see ref (30). In the spectrum for the ethanol-air interface, the narrow 3400 cm^–1^ signal is absent. As such, we assign this band for magnesite-ethanol to the OH groups of ethanol in the first layer, which apparently have a relative weak hydrogen-bonding interaction with the surface as the frequency is relatively high.^31^ The smaller full width half-maximum of this signal at 3400 cm^–1^, compared to the signal at 3200 cm^–1^, could point to a higher degree of order in this first layer; the OH groups of ethanol all seem to have a similar strength of hydrogen bonding with the magnesite surface. We assign the 3200 cm^–1^ band in the spectrum for the magnesite-ethanol interface to OH groups from ethanol molecules in the subsequent layers, dominated by the second layer as the orientation is opposite to the first layer and/or coming from a bulk contribution due to the interfacial charges breaking the centrosymmetry in the bulk ethanol. As the 3200 cm^–1^ signal is relatively strong, and it is known from the MD and AFM results that the ordering in the subsequent layers is relatively low, this signal might be dominated by the breaking of the centrosymmetry in the bulk of ethanol due to the charged interface. Under this assumption, the negative amplitude (with respect to the positive band at 3400 cm^–1^ being assigned to OH groups pointing with the H to the mineral) of this band suggests that the magnesite surface is positively charged.^24^

The calcite-ethanol interface also exhibits a double-peak SFG feature in the OH stretch region (Figure 3c), but the 3400 cm^–1^ signal is weak compared to that for the magnesite-ethanol interface (Figure 3d). In line with the magnesite-ethanol interface, the 3400 cm^–1^ signal is assigned to the ethanol in the first layer. The frequency of this band seems to be red-shifted for the calcite-ethanol interface compared to the magnesite-ethanol interface. This shift is in agreement with the MD simulations showing a shorter distance between the oxygen of the mineral carbonate group and the oxygen of ethanol for calcite compared to magnesite as clear from the vertical slices in Figure 2 and the histogram in Figure 4d. Moreover, the histograms for the oxygen–oxygen distance obtained from the MD simulations indicate that the hydrogen bonds between ethanol and the mineral are stronger than those between water and the mineral. The interaction between calcite and water seems to be slightly weaker than that for magnesite and water.

The 3200 cm^–1^ signal for the calcite-ethanol interface is, in line with the assignment for magnesite-ethanol, probably dominated by the bulk ethanol as the calcite interface might be slightly charged. Please note that this signal is ten times weaker than that for magnesite, indicating that the surface charge is probably also much smaller. The presence of this small surface charge for the calcite-ethanol interface raises the question of why it is not present for the calcite-water interface. We speculate that the 3200 cm^–1^ signal is not observed for the calcite-water interface, as the calcite slightly dissolves in water neutralizing the interface or producing ions that screen the surface charge (i.e., “self-screening”) and thus diminishing the electrostatically driven alignment of the water molecules. This idea is in line with AFM data indicating rapid self-cleaning (i.e., dissolution of the first layer) for calcite in water, which is less evident for magnesite. The very small signal for the calcite-water interface at 3400 cm^–1^ is assigned to the water molecules in the first and second hydration layer having opposite orientation and similar vibrational frequency and thus basically canceling out the signal. The similar vibrational frequency for the oppositely oriented water molecules is in line with the MD simulations showing only a small difference (by about 0.05 Å) in the oxygen–oxygen distance between carbonate and water and between water and water.

In summary, all the SFG data can be consistently explained in the following way. First, magnesite carries a significant surface charge, whereas calcite seems to carry a negligible surface charge. The SFG signal from the charged induced symmetry breaking of water in the bulk overwhelms the signal from the interfacial region for the water-magnesite interface. Second, in aqueous media, the SFG signals are consistently smaller compared to that in ethanol, which can be rationalized by neutralizing the surface due to dissolution or by self-screening of the surface charge due to the dissolved ions produced in the dissolution. This agrees with dissolution experiments of calcite in water and ethanol, demonstrating that calcite dissolution stops in the presence of ethanol.^32,33^ Third, the 3400 cm^–1^ signal, clearly observed for ethanol in contact with calcite and magnesite, indicates the presence of one ordered layer of OH groups from ethanol pointing to the mineral interface. The much smaller 3400 cm^–1^ signal for water in contact with calcite, demonstrates that in the water-calcite case, the first and second layers have opposing signals of equal frequency and thus equal strength of hydrogen bonding, canceling each other out. As the O–H stretch vibrational frequency provides information about the hydrogen-bonding strength,^31^ it can be concluded from the very weak, but present signal at a frequency of 3400 cm^–1^ that the O–H groups form a medium-strength hydrogen bond.^34^ In the case of ethanol, the peak resembles the hydrogen bond between the O–H group of ethanol and the substrate. This H-bond strength with the interface is clearly stronger than what is observed for water interacting with silica where the signal is observed at roughly 3660 cm^–1^.^35^

## Supporting Information Available

Description of AFM and SFG measurements and MD simulations, details of peak fitting, additional experiments with added salt, XPS results (PDF)
