# Supplementary material for: Water Orientation at the Calcite-Water Interface
Source: J Phys Chem Lett. 2021 Aug 5;12(31):7605–11. doi: 10.1021/acs.jpclett.1c01729 (PMC8365774; doi:10.1021/acs.jpclett.1c01729)
Supplement: Supplementary file 1 — jz1c01729_si_001.pdf [file jz1c01729_si_001.pdf]

## Supporting Information –

## „Water Orientation at the Calcite-Water Interface“

Hagen Söngen,[a] Simon J. Schlegel,[b] Ygor Morais Jaques,[d] John Tracey,[d] Saman Hosseinpour,[b*] Doyk Hwang,[b] Ralf Bechstein,[a] Mischa Bonn,[b] Adam S. Foster,[d,e] Angelika Kühnle,[a] Ellen H. G. Backus,[b,c]

[a]*Physical Chemistry I, Faculty of Chemistry, Bielefeld University, Universitätsstraße 25, 33615 Bielefeld, Germany*

[b]*Max Planck Institute for Polymer Research, Ackermannweg 10, 55128 Mainz, Germany*

[c]*Department of Physical Chemistry, University of Vienna, Währinger Strasse 42, 1090 Vienna, Austria*

[d]*Department of Applied Physics, Aalto University, Helsinki, FI-00076, Finland*

[e]*Nano Life Science Institute (WPI-NanoLSI), Kanazawa University, Kanazawa 920-1192, Japan*

* Present Address: Institute of Particle Technology (LFG), Friedrich-Alexander-Universitat-Erlangen-Nürnberg (FAU), Cauerstrasse 4, 91058 Erlangen, Germany

# 1 Methods

## 1.1 Atomic force microscopy

AFM experiments were performed using a modified commerical AFM setup (1) with photothermal excitation (2) and a custom 3D data acquisition system (3). Cuboid calcite and magnesite crystals (4x4 mm, Korth Kristalle GmbH) were cleaved along their (10.4) plane prior to each measurement. Measurement in pure water (MilliPore) were performed as described in previous publications (4,5). Measurements in ethanol have been described in a previous publication (6), from which we include the same experimental data.

## 1.2 Molecular dynamics simulations

The mineral slabs in ethanol as well as the simulation parameters, such as equilibration times and forcefields, are the same as those in reference 6. Additionally, we performed molecular dynamics of calcite and magnesite in water. For those simulations, we added 1000 water molecules to a simulation box with either calcite or magnesite. As in the other simulations, the box size was increased in order to accommodate the liquid molecules. We used the flexible water model SPC/Fw. (7)

The simulations were performed with LAMMPS (8), using the velocity Verlet algorithm (9) as the integrator. Electrostatics were calculated with the particle-particle-particle-mesh (P3M) method. (10) The simulations were performed at 300 K with Nosé-Hoover thermostats (11–13) with a damping factor of 0.1 ps. Nosé-Hoover barostats (11) were used only in one stage of the equilibration, in the direction perpendicular to the surface, at 1 atm and with a damping factor of 1 ps. We used a timestep of 1 fs for the simulations.

Hydrogen bond and dipole orientation analysis were done with the MDAnalysis (14) software. The criteria utilized to identify hydrogen bonds were: a maximum distance of 0.3 nm between water oxygen and carbonate oxygen (donor and acceptor atoms respectively) and a donor-hydrogen-acceptor angle of 150 degrees. The same criteria were used in the ethanol simulations, where the donor was the oxygen from the hydroxy group. The analyses were conducted in the production run of 10 ns after the equilibration.

## 1.3 Sum frequency generation spectroscopy

For the SFG spectroscopy the setup described in reference (15) was used. The angle of the incoming visible and infrared light was 56° and 40° with the surface normal, respectively. (15)

As samples we used a polished single-crystal magnesite window (square shape, 12x12 mm, thickness 1.5 mm) from SurfaceNet GmbH and a polished single-crystal calcite window (circular shape, diameter 25 mm, thickness 2 mm) from Korth Kristalle GmbH. For the SFG experiments the samples have to be polished as otherwise the light will scatter. Therefore, unfortunately, we could

not use cleaved substrates which is the more common preparation method for these materials. Both samples are uniaxially birefringent and have been cut such that the (10.4) plane is exposed on both the top and the bottom interface. The orientation of the optical axis was determined by identifying the projection of the $[42\bar{1}]$ direction on the (10.4) surface of the crystals, as described in reference (16): When looking perpendicular to the (10.4) surface through the mineral, any feature imaged along the extraordinary direction is displaced along $[42\bar{1}]$. This allowed us to make sure that the experiments were indeed carried out at the mineral–water interface, not the mineral–air interfaces. The plane of incidence of the incoming light was chosen to be aligned with the optical axis (antiparallel to the $[42\bar{1}]$ surface direction). The incoming visible and infrared beam travel through the mineral. The SFG light is generated at the mineral-liquid interface and travels again through the mineral.

We used the same ethanol as we used in the AFM experiments (Sigma Aldrich, article number 32205, purity ≥ 99.8). In contrast to the AFM experiments, where we used new samples in each measurement session, we re-used the SFG samples and cleaned them after each measurement with pure water (Millipore) and using an Ozone/$O_2$ cleaner for 20 min.

For calcite-water interfaces, the acquisition time was typically chosen to be one hour (3600 s). All other sample systems were measured with shorter acquisition time in the order of minutes.

All spectra presented in this manuscript are background-corrected SFG intensity spectra. All spectra are normalized with respect to the acquisition time. In most cases, an additional normalization has been performed using a reference spectrum obtained from the mineral-gold interface of a gold-coated magnesite and a gold-coated calcite sample, respectively.

While the calcite sample was optically clear, the magnesite sample had visible cracks and optical impurities (due to the fact that it is a natural sample, as opposed to the synthetic calcite). Therefore, the absolute intensity of the magnesite spectra varied greately (by approximately a factor in the order of 5, depending on where the visible and infrared light travelled through the crystal).

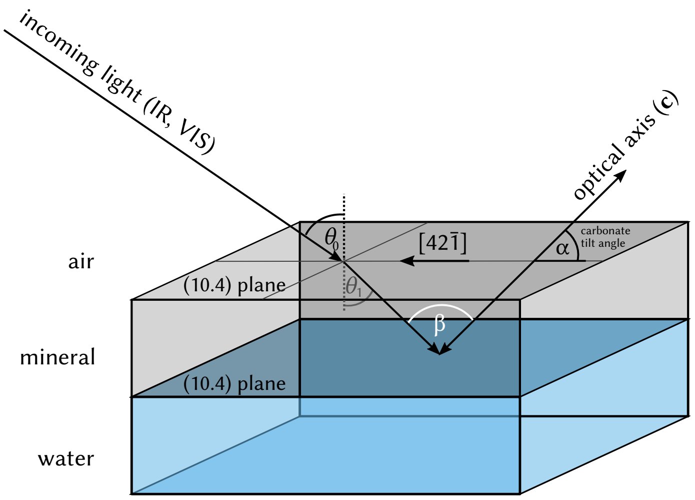


Supporting Figure S 1: Relation between the incoming light (angle $\theta$ with the surface normal) and the optical **c**-axis (angle $\alpha \approx 44°$ with the surface plane). The resulting angle $\beta = \theta_1 + 90° - \alpha$ between the incident light and the **c**-axis is important for identifying the refractive index inside the birefringent minerals for different polarizations.

### 1.3.1 Crystal orientation in SFG experiments

Both calcite and magnesite are minerals with a symmetry described by the space group R$\bar{3}$c. (17) Both minerals are uniaxially birefringent. The optical axis is the **c**-axis, which is oriented perpendicular to the plane spanned by the oxygen atoms of the carbonate groups. (16, 17) Thus, the projection of the **c**-axis on the (10.4) surface is aligned with the [42$\bar{1}$] direction (see Figure S 1). The angle between the **c**-axis and the (10.4) surface is approximately 44° (which is the „tilt angle“ of the carbonate groups).

We used mineral samples with an exposed (10.4) plane on both the top and the bottom interface (see Figure S 1). The orientation of the **c**-axis was determined by identifying the [42$\bar{1}$] direction on the (10.4) surface of the crystals, as described in reference 16: When looking perpendicular to the (10.4) surface through the mineral, any feature imaged along the extraordinary direction is displaced along [42$\bar{1}$]. This identification is summarized in Figure S 2.

The incoming visible and infrared lightbeams have to travel through the mineral to reach the mineral–water interface. Depending on the polarization of both the visible and the infrared lights, the speeds of the two incoming rays can be different. In the most general case, where the **c**-axis is

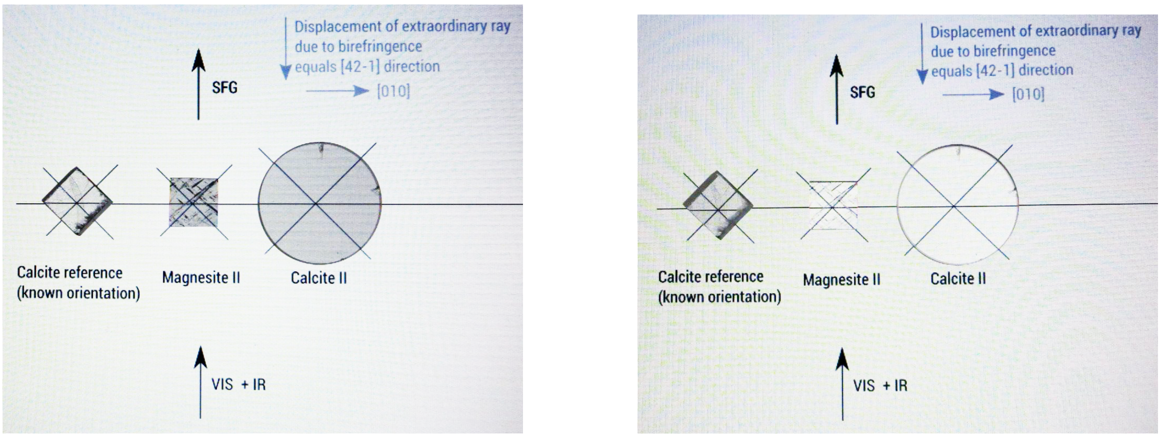


Supporting Figure S 2: Top view on calcite and magnesite crystals through a polarization filter. The „calcite reference“ is a thick calcite mineral where the determination of the orientation is straightforward. Additionally, the calcite mineral and the magnesite mineral used in the experiment are shown. In the image on the left, only light traveling perpendicular to the optical axis is shown (ordinary refraction, no displacement). On the right photograph, only light traveling parallel to the optical axis is shown. The right photograph shows a clear displacement in the $[42\bar{1}]$ direction.

arbitrarily aligned with respect to the incoming light, the polarization plane of the incoming light can be changed (as in a wave retarder). The polarization of the incoming light is maintained only if the optical axis lies within the plane of incidence. This still gives two possible sample orientations: One as shown in Figure S 1 and another one where the mineral is rotated by 180° in the surface plane. These two possible sample orientations are detailed in the next two paragraphs.

**Incoming light perpendicular to the optical axis** First, we discuss the case used in the experiment: The incoming light propagates in a direction perpendicular to the optical axis. If the incoming light is s-polarized, the electric field vector is perpendicular to the optical axis. The light travels through the mineral according to the principal ordinary refractive index $\bar{n}_o$:

$$n_o(\beta) = \bar{n}_o \tag{1}$$

If the incoming light is p-polarized, the polarization is approximately parallel to the optical axis of calcite and magnesite. In this case, the light travels through the mineral according to a refractive

index that depends on the angle $\beta$ between the incident ray and the optical axis according to[1]

$$n_e(\beta) = \sqrt{\left(\frac{\cos^2\beta}{\bar{n}_o^2} + \frac{\sin^2\beta}{\bar{n}_e^2}\right)^{-1}}, \tag{2}$$

where $\bar{n}_e$ is the principal extraordinary refractive index.

In the experiments, the crystals were mounted so that the angle between incoming light is approximately perpendicular to the optical axis ($\beta \approx 90°$), with the optical axis inside the plane of incidence. For $\beta = 90°$, p-polarized incoming light travels through the mineral according to $\bar{n}_e$.

Thus, the speed of light inside the medium is different for s-polarized and p-polarized light. In the experiment, the SSP polarization combination was used, meaning that the outgoing SFG light and the incoming visible light are s-polarized, whereas the incoming infrared light is p-polarized. Should the temporal and spatial focal point of the incoming visible and infrared beams have been (mistakenly) placed at the mineral–air interface (the upper interface), the speed of the two rays is equal, regardless of their polarization. When choosing the focal point at the mineral–water interface (the lower interface), a change in the delay (of the infrared light relative to the visible light) is expected when changing the polarization of the visible light from s to p. This was used to experimentally confirm that indeed the mineral–water interface (and not the mineral–air interface) was probed, as is detailed in the following.

The group velocities $v$ (the ratio between the speed of light $c$ and the refractive index inside a medium) of the ordinary and the extraordinary ray in calcite and magnesite are:

$$v_o^{Ca} = 182 \times 10^6\,\mathrm{m\,s^{-1}} \qquad v_o^{Mg} = 175 \times 10^6\,\mathrm{m\,s^{-1}} \tag{3}$$

$$v_e^{Ca} = 202 \times 10^6\,\mathrm{m\,s^{-1}} \qquad v_e^{Mg} = 199 \times 10^6\,\mathrm{m\,s^{-1}} \tag{4}$$

The distance traveled by light passing through the crystals is

$$\Delta s = h/\cos\theta_1 \tag{5}$$

[1]Equation 6.3-15 in reference 18

where $\theta_1$ is the angle in the mineral (measured relative to the surface normal) and $h$ is the thickness of the mineral ($h = 2\,\mathrm{mm}$ for calcite and $h = 1.5\,\mathrm{mm}$ for magnesite).[2] The timing difference $\Delta t = \Delta s/v_\mathrm{o} - \Delta s/v_\mathrm{e}$ for passing through the mineral is

$$\Delta t^{\mathrm{Ca}} = 1.71\,\mathrm{ps} \qquad \Delta t^{\mathrm{Mg}} = 1.35\,\mathrm{ps}. \tag{6}$$

In the experiments, the displacement of the delay stage $\Delta d = c\Delta t/2$ is adjusted:

$$\Delta d^{\mathrm{Ca}} = 0.256\,\mathrm{mm} \qquad \Delta d^{\mathrm{Mg}}_{\mathrm{delay}} = 0.203\,\mathrm{mm} \tag{7}$$

For calcite a delay difference of 0.215 mm and for magnesite a delay difference of 0.160 mm was obtained when switching the polarization of the visible beam from P to S (*i.e.*, switching from PPP to SSP). The small discrepancy with respect to the calculated values might arise from an additional delay introduced by the polarizer. Should the infrared and visible light be focused on the mineral–air boundary (i.e. the upper boundary), no change in delay would be expected when switching from SSP to PPP.

**Incoming light parallel to the optical axis** For illustrative purposes, let us assume the crystals were mounted so that the angle $\beta$ between the incoming light and the optical axis is approximately zero (this is *not* the orientation used in the experiments). If the incoming light is s-polarized, the polarization is perpendicular to the optical axis. If the incoming light is p-polarized, the polarization is also perpendicular to the optical axis.

In both cases, the light travels through the mineral according to the „ordinary“ refractive index $n_\mathrm{o}$. Importantly, the speed of light inside the medium is equal for s-polarized and p-polarized light. For a mineral orientation rotated by 180°, this behavior was confirmed: no delay change was necessary when switching from PPP to SSP.

[2] The angle $\theta_1$ is obtained by numerically solving $\theta_1 = \arcsin(\sin(\theta) \cdot n_0/n_1)$ and equation (2) with $\beta = \theta + \pi/2 - \alpha$.

### 1.3.2 Fresnel factors

The angle of incidence at the mineral–air boundary is $\theta$, the angle of incidence at the mineral–water interface is $\theta_2$, with

$$\theta_1 = \arcsin\left(\frac{n_0}{n_1}\sin\theta\right) \tag{8}$$

$$\theta_2 = \arcsin\left(\frac{n_1}{n_2}\sin\theta_1\right) \tag{9}$$

where $n_0$ is the refractive index of air, $n_1$ the refractive index of the mineral and $n_2$ the refractive index of water (or gold). The $L$-factors for a planar two-medium system are (19)

$$L_{xx} = \frac{2n_1\cos\theta_2}{n_2\cos\theta_1 + n_1\cos\theta_2} \tag{10}$$

$$L_{yy} = \frac{2n_1\cos\theta_1}{n_1\cos\theta_1 + n_2\cos\theta_2} \tag{11}$$

$$L_{zz} = \frac{2n_2\cos\theta_1}{n_1\cos\theta_2 + n_2\cos\theta_1}\left(\frac{n_1}{n'}\right)^2 \tag{12}$$

where it was assumed that the interfacial refractive index $n'$ is given by the refractive index of the second medium, i.e. water or ethanol. As the mineral surfaces are not isotropic, measurements using SSP polarization combination are sensitive to the $yyz$ and $yyx$ elements. Here, we assume that the influence of the anisotropy is small and may not even be projected on the solvent molecules. Thus, for simplicity we consider here in the discussion only the $yyz$ component. Under this assumption, the SFG intensity for the SSP polarization combination is proportional to

$$I_{\text{ssp}} \propto |L_{yy}(\lambda_{\text{SFG}})L_{yy}(\lambda_{\text{VIS}})L_{zz}(\lambda_{\text{IR}})|^2 \,. \tag{13}$$

For computation of $L_{yy}$ for calcite and magnesite, the index of refraction for the ordinary ray ($o$) is used. For $L_{zz}$, the index of refraction for the extraordinary ray ($e$) is used. For the SFG beam, an angle of $\theta = 50°$ was used, for VIS an angle of 56° was used and for the IR light an angle of 40° was used. (15) The (complex) indices of refraction were looked up from different sources and are summarized in table S 1: Data from calcite is used according to Ghosh. (20) Unfortunately, no

| | Calcite *o* | Calcite *e* | Magnesite *o* | Magnesite *e* | Gold | Water | Ethanol |
|---|---|---|---|---|---|---|---|
| $\Re n(\lambda_{SFG})$ | 1.6548 | 1.4845 | 1.710 | 1.509 | 0.1546 | 1.331 | 1.3595 |
| $\Im n(\lambda_{SFG})$ | 0 | 0 | 0 | 0 | 3.647 | $1.64 \times 10^{-8}$ | $8.514 \times 10^{-8}$ |
| $\Re n(\lambda_{VIS})$ | 1.6488 | 1.4819 | 1.710 | 1.509 | 0.1244 | 1.329 | 1.3565 |
| $\Im n(\lambda_{VIS})$ | 0 | 0 | 0 | 0 | 5.004 | $1.25 \times 10^{-7}$ | $1.6085 \times 10^{-7}$ |
| $\Re n(\lambda_{IR})$ | 1.6209 | 1.4761 | 1.710 | 1.509 | 1.358 | 1.450 | 1.5163 |
| $\Im n(\lambda_{IR})$ | 0 | 0 | 0 | 0 | 22.91 | 0.0368 | 0.066 210 |

Supporting Table S 1: Complex refractive index for all materials considered.

index of refraction for a wavelength larger than 2170 nm has been found in literature. Therefore, a value of $n$ for a wavelength of 2170 nm is used for the IR wavelength of 3333 nm. This assumption is corroborated by an index of refraction that is insensitive to the wavelength in the region of $\lambda > 2500$ nm. (21) For magnesite, a wavelength-independent index of refraction was used. (17) The (complex) indices of refraction for gold (22) and water (23) were taken from literature as well.

The results for the proportionality factor $I_{ssp}$ is presented below for the mineral–liquid interfaces presented here, as well as for the mineral–gold interfaces used for normalization. Importantly, the order of magnitude is similar for all cases.

$$I_{ssp}^{Ca/H_2O} = 1.9, \qquad I_{ssp}^{Ca/Au} = 11 \times 10^{-6}, \qquad I_{ssp}^{Ca/H_2O}/I_{ssp}^{Ca/Au} = 182 \times 10^{3} \tag{14}$$

$$I_{ssp}^{Ca/eth} = 1.6, \qquad I_{ssp}^{Ca/Au} = 11 \times 10^{-6}, \qquad I_{ssp}^{Ca/eth}/I_{ssp}^{Ca/Au} = 149 \times 10^{3} \tag{15}$$

$$I_{ssp}^{Mg/H_2O} = 2.2, \qquad I_{ssp}^{Mg/Au} = 14 \times 10^{-6}, \qquad I_{ssp}^{Mg/H_2O}/I_{ssp}^{Mg/Au} = 164 \times 10^{3} \tag{16}$$

### 1.3.3 Peak fitting

We fitted a simplified model for the complex second-order susceptibility $\chi^{(2)}$ to our experimental intensity data using the following expression: (24)

$$I \propto |\chi^{(2)}|^2 = \left| A_0 e^{i\varphi_0} + \frac{A_1}{\tilde{\nu}_1 - \tilde{\nu} + i\Gamma_1} + \frac{A_2}{\tilde{\nu}_2 - \tilde{\nu} + i\Gamma_2} \right|^2 \tag{17}$$

This model includes a wavenumber-independent non-resonant contribution $A_0 e^{i\varphi_0}$ and two resonant (Lorentzian) peaks centered around the wavenumbers $\nu_1$ and $\nu_2$ with a full width at half maximum of $2\Gamma_1$ and $2\Gamma_2$ in the intensity spectrum, respectively.

In the resulting fit for the magnesite–ethanol interface (Fig. S 3), the wavenumbers are 3220 cm$^{-1}$ and 3420 cm$^{-1}$. The phase shift of the fitted non-resonant signal is -53°.

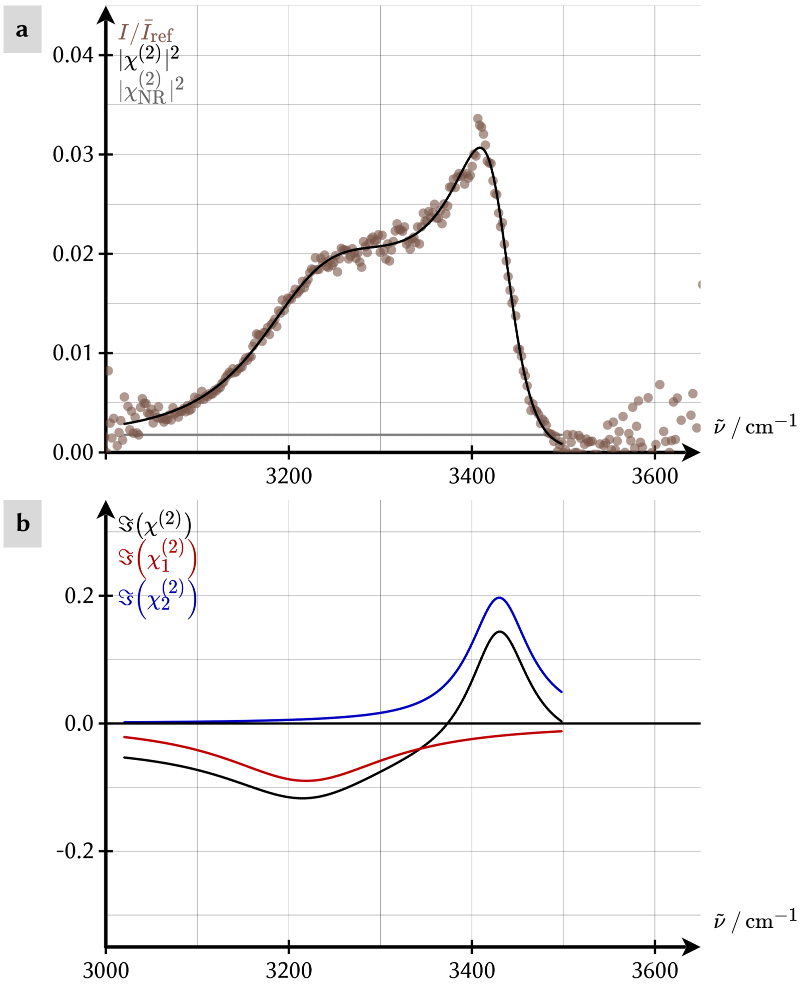


Supporting Figure S 3: (a) SFG intensity spectrum for the magnesite-ethanol interface together with the fit (black line) with the Lorentzian lineshape model (see above). The grey line shows the contribution in the fit of the nonresonant signal. (b) Resulting $\Im(\chi^{(2)})$ spectrum from the fit (black line) together with the individual resonances (red and blue lines).

# 2 Additional data

## 2.1 SFG spectra for aqueous sodium chloride solutions

### 2.1.1 Calcite

To exclude that the absence of the signal for pure water in contact with calcite is due to interference effects as explained in Ref. 25, we have performed an SFG experiment with 1 mM NaCl solution. Clearly, Figure S 4 shows that also at 1 mM NaCl solution the signal is still absent. As such, we conclude that the absence of the signal for pure water is not caused by interference effects.

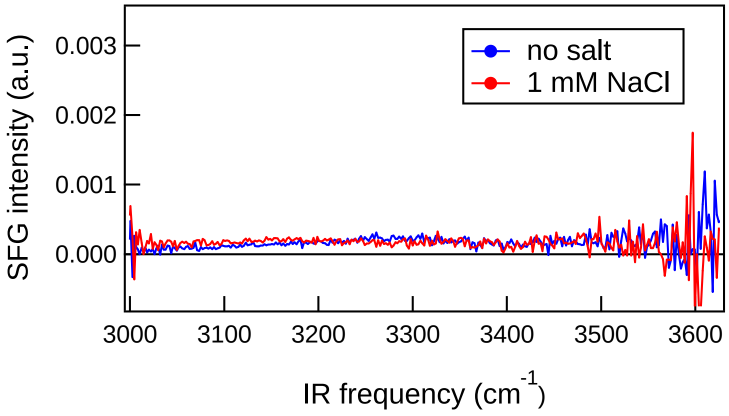


Supporting Figure S 4: SFG intensity for the calcite water interface in ssp polarization as function of the IR frequency for pure water and water with 1 mM NaCl.

### 2.1.2 Magnesite

Additional SFG spectra taken at the magnesite-water interface at different concentrations of sodium chloride are shown in Figure S 5.

## 2.2 Comment on SFG measurements with PPP polarization

A very small signal at high frequency ($\approx 3500\ \mathrm{cm}^{-1}$) has been observed for the water-calcite interface with PPP polarization.

The SSP signal is determined by the $yyz$ tensor element with a possible contribution of the $yyx$ element as the (10.4) surface of calcite and magnesite are anisotropic. However, if the anisotropy

is not projected on the solvent molecules, the signal is solely determined by the $yyz$ element. This is certainly the case for the bulk contribution to the signal due to the surface charge.

The PPP signal is governed by the $xxz$, $xzx$, $zxx$, and $zzz$ tensor elements with additional contributions of the $xxx$, $xzz$, $zxz$ and $zzx$ elements due to the anisotropy. As such, the PPP signal is not straightforward to interpret, and might have contributions from both the out-of-plane projection ($z$-component of the infrared response) and the in-plane projection ($x$-component of the infrared response) of the transition dipole moment.

The SSP signal is dominated by the out-of-plane projection ($z$-component of the infrared response) of the transition dipole moment.

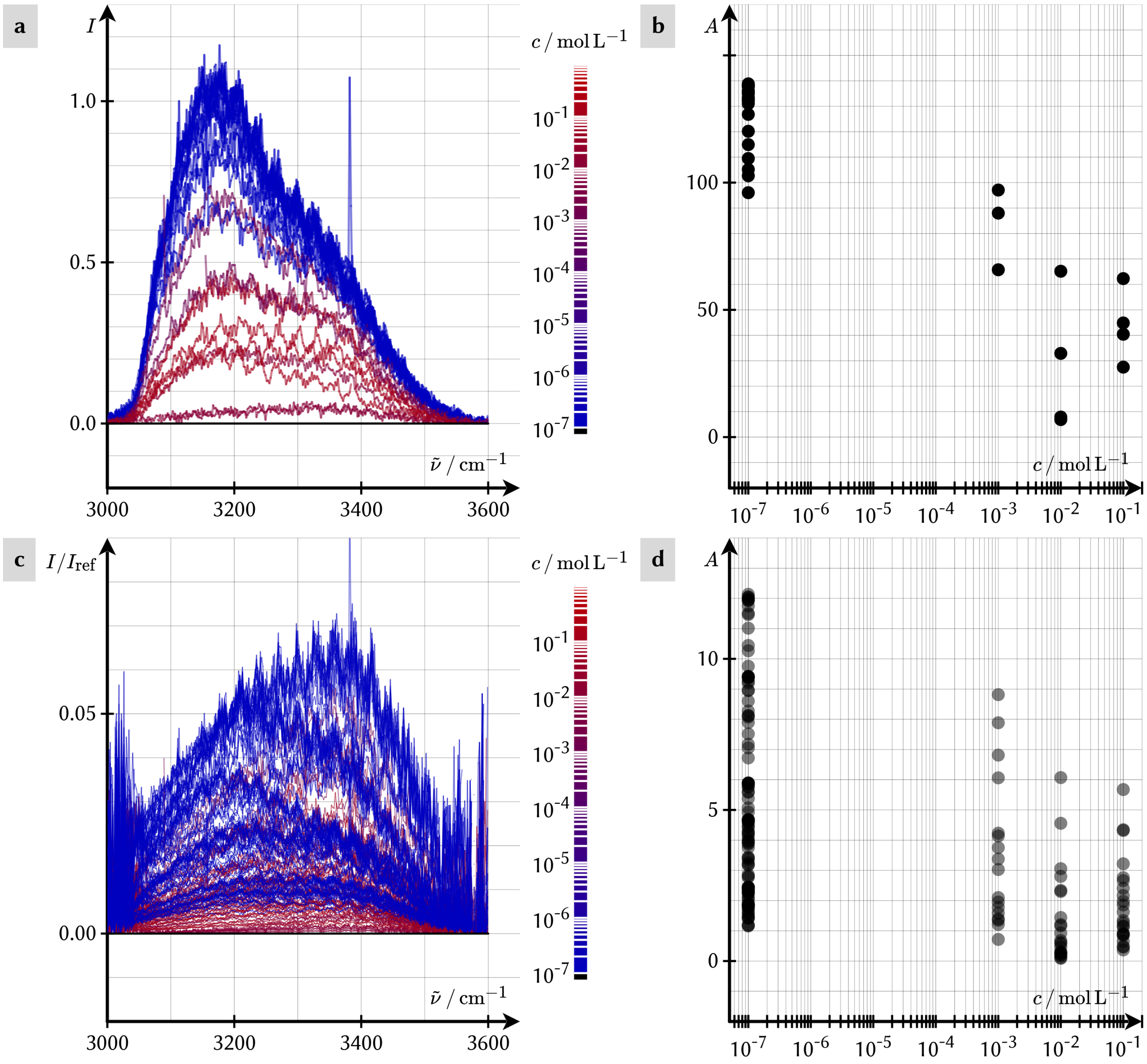


Supporting Figure S 5: Comparison of SFG intensity spectra of magnesite-water interfaces at different concentrations of sodium chloride. (a) SFG spectra (without normalization for the IR lineshape) for the magnesite-water interface with varying NaCl ionic strength in the aqueous phase. (b) SFG intensity integrated from 3000 cm$^{-1}$ to 3600 cm$^{-1}$ as a function of NaCl concentration. (c and d) Same data as in panel a and b but normalized for the IR lineshape.

## 2.3 XPS

The XPS spectra for the O 1s of the magnesite sample (Figure S 6 (a)) shows much stronger additional peaks at the low binding energy side of the main O 1s peak (marked by an asterisks in the figure) than is the case in the O 1s of the calcite sample (Figure S 6 (b)), indicating that the magnesite is likely less pure than the calcite. This conclusion is supported by a change in the peak shape of the Mg 2p at different sample positions, specifically the change in the tail on the low binding energy side of the Mg 2p (Figure S 6 (c)) signal of magnesite. In comparison the Ca 2s region does not show any additional peaks and shows a consistent Ca 2s peak shape at all three sample positions (Figure S 6 (d)).

The binding energy of the XPS data was aligned using the O1 s binding energy of calcium for calcite and the O1s binding energy of magnesium for magnesite, with values taken from reference 26.

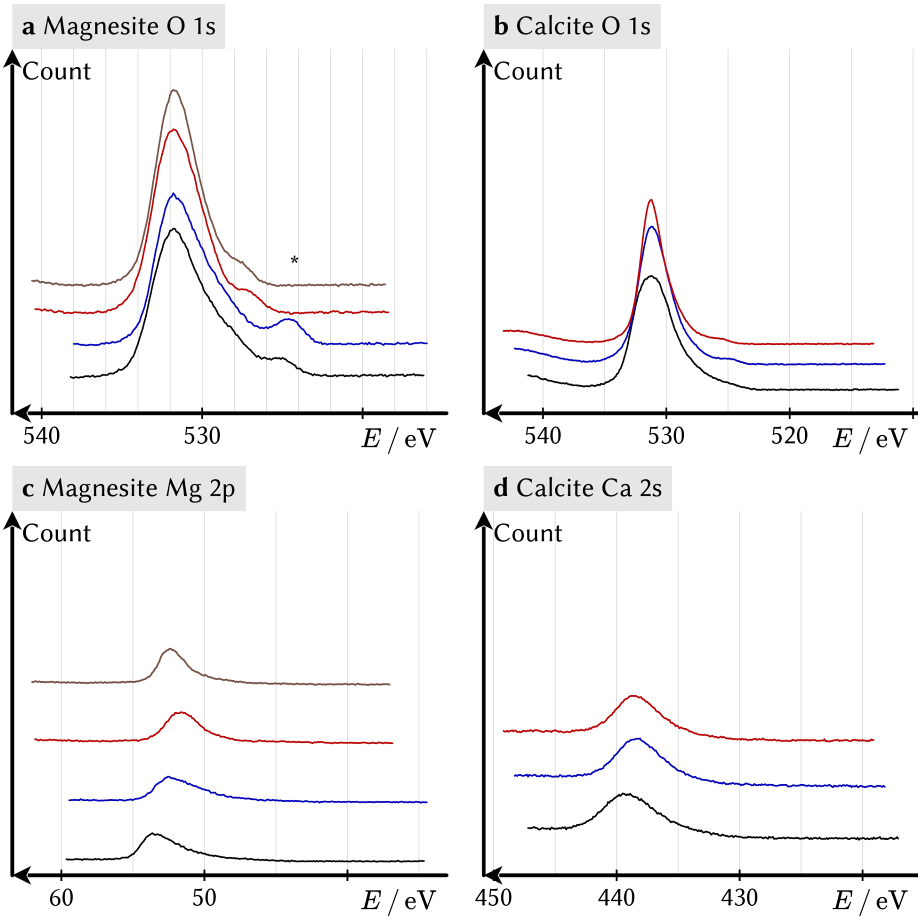


Supporting Figure S 6: XPS spectra for the O 1s orbital of (a) magnesite and (b) calcite, (c) the Mg 2p orbital of magnesite, and (d) the Ca 2s orbital of calcite at different positions on the sample. Data for the various measurements is offset vertically.

## References

[1] S. Rode, M. Schreiber, A. Kühnle, and P. Rahe. Frequency-modulated atomic force microscopy operation by imaging at the frequency shift minimum: the dip-df mode. *Rev. Sci. Instrum.*, 85(4):043707, 2014.

[2] H. Adam, S. Rode, M. Schreiber, K. Kobayashi, H. Yamada, and A. Kühnle. Photothermal Excitation Setup for a Modified Commercial Atomic Force Microscope. *Rev. Sci. Instrum.*, 85(2):23703, 2014.

[3] H. Söngen, M. Nalbach, H. Adam, and A. Kühnle. Three-Dimensional Atomic Force Mi-

croscopy Mapping at the Solid-Liquid Interface with Fast and Flexible Data Acquisition. *Rev. Sci. Instrum.*, 87(6):063704, 2016.

[4] H. Söngen, C. Marutschke, P. Spijker, E. Holmgren, I. Hermes, R. Bechstein, S. Klassen, J. Tracey, A. S. Foster, and A. Kühnle. Chemical Identification at the Solid–Liquid Interface. *Langmuir*, 33(1):125–129, 2017.

[5] H. Söngen. Atomic Force Microscopy at Mineral–Water Interfaces: Hydration, Chemical Identification and Point Defects, 2018.

[6] H. Söngen, Y. M. Jaques, P. Spijker, C. Marutschke, S. Klassen, Hermes, I., R. Bechstein, L. Zivanovic, J. Tracey, A. S. Foster, and A. Kühnle. *Beilstein J. Nanotechnol.*, 11:891–898, 2020.

[7] Y. Wu, H. L. Tepper, and G. A. Voth. Flexible simple point-charge water model with improved liquid-state properties. *J. Chem. Phys.*, 124(2):024503, 2006.

[8] S. Plimpton. Fast Parallel Algorithms for Short-Range Molecular Dynamics. *J. Comput. Phys.*, 117(1):1–19, 1995.

[9] W. C. Swope, H. C. Andersen, P. H. Berens, and K. R. Wilson. A computer simulation method for the calculation of equilibrium constants for the formation of physical clusters of molecules: Application to small water clusters. *J. Chem. Phys*, 76(1):637, 1982.

[10] Roger W. Hockney and James W. Eastwood. *Computer simulation using particles.* A. Hilger, Bristol [England] ; Philadelphia, special student ed edition, 1988.

[11] Wataru Shinoda, Motoyuki Shiga, and Masuhiro Mikami. Rapid estimation of elastic constants by molecular dynamics simulation under constant stress. *Phys. Rev. B*, 69:134103, Apr 2004.

[12] S. Nosé. A unified formulation of the constant temperature molecular dynamics methods. *J. Chem. Phys*, 81(1):511–519, 1984.

[13] W. G. Hoover. Canonical dynamics: Equilibrium phase-space distributions. *Phys. Rev. A*, 31(3):1695–1697, 1985.

[14] N. Michaud-Agrawal, E. J. Denning, T. B. Woolf, and O. Beckstein. MDAnalysis: A toolkit for the analysis of molecular dynamics simulations. *J. Comput. Chem.*, 32(10):2319–2327, 2011.

[15] S. Hosseinpour, F. Tang, F. Wang, R. A. Livingstone, S. J. Schlegel, T. Ohto, M. Bonn, Y. Nagata, and E. H. G. Backus. Chemisorbed and Physisorbed Water at the $TiO_2$/Water Interface. *J. Phys. Chem. Lett.*, 8(10):2195–2199, 2017.

[16] S. Kuhn, M. Kittelmann, Y. Sugimoto, M. Abe, A. Kühnle, and P. Rahe. Identifying the absolute orientation of a low-symmetry surface in real space. *Phys. Rev. B.*, 90(19):195405, 2014.

[17] W. A. Deer, R. A. Howie, and J. Zussman. *An introduction to the rock-forming minerals.* 2nd edition, 1992.

[18] B. E. A. Saleh and M. C. Teich. *Fundamentals of Photonics.* Wiley, 1991.

[19] X. Zhuang, P. B. Miranda, D. Kim, and Y. R. Shen. Mapping molecular orientation and conformation at interfaces by surface nonlinear optics. *Phys. Rev. B*, 59(19):12632–12640, 1999.

[20] G. Ghosh. Dispersion-equation coefficients for the refractive index and birefringence of calcite and quartz crystals. *Opt. Commun.*, 163:95–102, 1999.

[21] L. L. Long, M. R. Querry, R. J. Bell, and R. W. Alexander. Optical properties of calcite and gypsum in crystalline and powdered form in the infrared and far-infrared. *Infrared Phys.*, 34(2):191–201, 1993.

[22] R. L. Olmon, B. Slovick, T. W. Johnson, D. Shelton, S.-H. Oh, G. D. Boreman, and M. B. Raschke. Optical dielectric function of gold. *Phys. Rev. B*, 86(23):235147, 2012.

[23] G. M. Hale and M. R. Querry. Optical Constants of Water in the 200-nm to 200-$\mu$m Wavelength Region. *Appl. Opt.*, 12(3):555, 1973.

[24] M. Sovago, E. Vartiainen, and M. Bonn. Determining Absolute Molecular Orientation at Interfaces: A Phase Retrieval Approach for Sum Frequency Generation Spectroscopy. *J. Phys. Chem. C*, 113(15):6100–6106, 2009.

[25] J. Schaefer, G. Gonella, M. Bonn, and E. H. G. Backus. Surface-specific vibrational spectroscopy of the water/silica interface: screening and interference. *Phys. Chem. Chem. Phys.*, 19:16875–16880, 2017.

[26] J. T. Kloprogge and B. J. Wood. Handbook of Mineral Spectroscopy. 2020.
